# A holstein heifer infected with *Neospora caninum* NcUru3 congenitally transmits this strain to a viable offspring although infection does not protect her from aborting by a different *N. caninum* genotype in the subsequent gestation

**DOI:** 10.3389/fvets.2022.889157

**Published:** 2022-07-25

**Authors:** Federico Giannitti, Virginia Aráoz, Caroline da Silva Silveira, María E. Francia, Carlos Robello, Andrés Cabrera

**Affiliations:** ^1^Plataforma de Investigación en Salud Animal, Instituto Nacional de Investigación Agropecuaria, Estación Experimental La Estanzuela, Colonia, Uruguay; ^2^Laboratorio de Biología de Apicomplejos, Institut Pasteur de Montevideo, Montevideo, Uruguay; ^3^Departamento de Parasitología y Micología, Facultad de Medicina, Universidad de la República, Montevideo, Uruguay; ^4^Laboratorio de Interacciones Hospedero-Patógeno, Instituto Pasteur de Montevideo, Montevideo, Uruguay; ^5^Departamento de Bioquímica, Facultad de Medicina, Universidad de la República, Montevideo, Uruguay; ^6^Unidad de Microbiología, Departamento de Patobiología, Facultad de Veterinaria, Universidad de la República, Montevideo, Uruguay

**Keywords:** abortion, dairy cattle, genetic diversity, *Neospora caninum*, multilocus microsatellite typing, molecular epidemiology, pathology, reproductive diseases

## Abstract

*Neospora caninum* is a leading cause of bovine abortion worldwide. Although the genetic diversity of this apicomplexan parasite has long been recognized, there is little information on whether infection with different genotypes results in different clinical outcomes or whether infection by a given genotype impairs protective immunity against abortion induced by different genotypes. Here, we provide evidence supporting that natural subclinical infection with isolate NcUru3 of *N. caninum* in a pregnant heifer did not provide protection against abortion caused by a different *N. caninum* genotype in the subsequent gestation. A Holstein heifer delivered a healthy calf congenitally infected with *N. caninum*. Specific anti-*N. caninum* IgG was detected by indirect ELISA in sera obtained from the dam at calving and the calf before ingestion of colostrum, indicating *in utero* exposure to the parasite in the latter. A *N. caninum* strain named NcUru3 was isolated and characterized by multilocus microsatellite typing from the brain of this neonate euthanized at 9 days of age. Sixty days after calving, the cow got pregnant, although she aborted spontaneously at ~6 months of gestation. Pathologic examination of the aborted fetus and placenta revealed typical lesions of neosporosis, including encephalitis, myocarditis, hepatitis, myositis, and placentitis. *Neospora caninum* DNA was amplified from the fetal brain, heart, kidney, and placenta, and multilocus microsatellite typing revealed a genotype that differed from isolate NcUru3 at the level of microsatellite marker 6A (MS6A). Serum obtained from the dam at the time of abortion had IgG that cross-recognized isolate NcUru3, as demonstrated by immunoblotting, indicating that the humoral immune response did not prevent the other genotype from infecting the fetus and inducing fetoplacental lesions and abortion. This is the first description of one same dam transmitting two *N. caninum* genotypes to her offspring in subsequent gestations.

## Introduction

The apicomplexan parasite *Neospora caninum* is one of the leading causes of abortion in cattle and results in significant economic losses to the livestock industry worldwide (1, 2). Multilocus microsatellite markers used to characterize *N. caninum* isolates showed extensive genetic diversity, implying recent genetic diversification from a common ancestor (3, 4). This hypothesis was further supported by a recent study in which 50 *N. caninum* strains from a range of hosts (dog, cattle, deer, horse, and rhinoceros) and geographic locations (North and South America, Europe, Asia, and Australia), were genotyped using 19 linked and unlinked genetic markers. The study concluded that a single highly inbred *N. caninum* genotype swept globally, likely through the global expansion of European cattle breeds (5). There is evidence that this clonal propagation in cattle led to population sub-structuring in different geographical regions throughout the globe, resulting in genetic diversity among *N. caninum* isolates (6, 7).

It is generally accepted that different *N. caninum* strains differ in virulence and pathogenicity, which may partially explain variations in disease epidemiology and clinical presentation in the field (8). However, there is a gap of knowledge regarding clinical outcomes in cattle infected with different *N. caninum* strains/genotypes or whether infection with a given strain would confer protection against abortion by other strains under natural circumstances. In this brief report, we provide evidence supporting that natural subclinical infection with isolate NcUru3 of *N. caninum* in a pregnant Holstein heifer did not provide protection against abortion caused by a different *N. caninum* genotype in the subsequent gestation in the same dam, which represents a relevant contribution to the current knowledge on the epidemiology of neosporosis in cattle.

## Methods, results, and discussion

On April 10, 2017, a nulliparous Holstein heifer of 25 months and 19 days of age from a dairy farm in Colonia, Uruguay delivered a clinically healthy male calf congenitally infected with *N. caninum* after natural exposure. Serum samples were obtained from the dam immediately after parturition and the newborn calf before colostrum ingestion. Both samples were processed by a commercial indirect ELISA for the detection of anti-*N. caninum* IgG (LSIVet bovine neosporosis advanced serum ELISA kit, Thermo Fisher Scientific, LSINEOA5), with positive results. Upon precolostral seropositivity, and following a procedure approved by INIA's animal ethics committee for the use of animals in experimentation (CEUA-INIA protocol # 2017.1), the calf was euthanized at 9 days of age. Immediately after euthanasia the brain was collected aseptically and processed for *N. caninum* culture; strain NcUru3 was isolated and further molecularly characterized by microsatellite typing as we previously reported (9).

**Figure 1:**
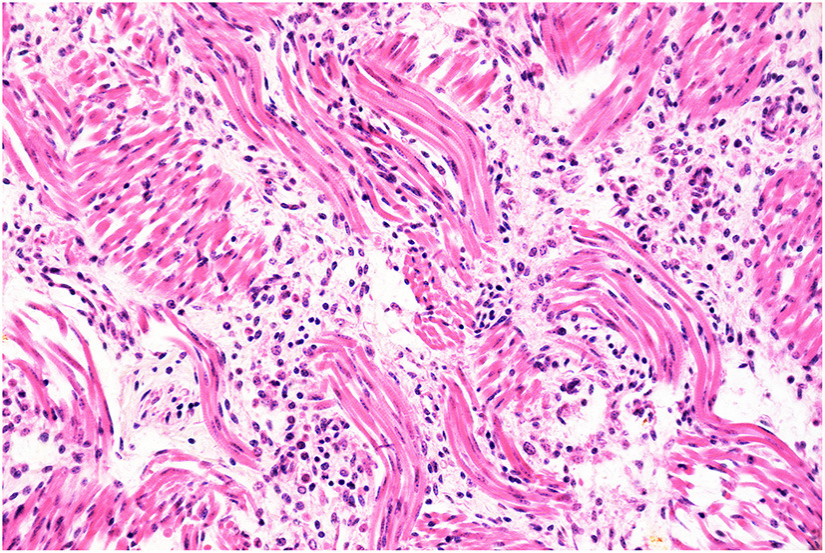
Histopathology of the tongue. Glossal myositis. The interstitium separating the myocytes is infiltrated by moderate numbers of inflammatory cells, notably lymphocytes and histiocytes. H&E stain, original magnification 400x.

**Figure 2:**
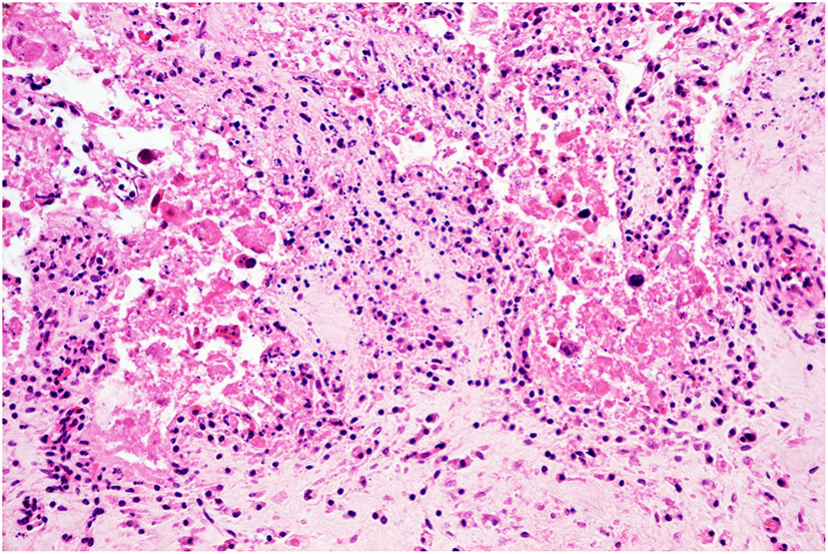
Histopathology of the placenta. Placentitis. The chorionic stroma is expanded by edema and infiltrated by moderate numbers of lymphocytes and histiocytes. The trophoblastic epithelium is effaced and replaced by hypereosinophilic and karyorrhectic sloughed cellular debris, indicating severe necrosis. H&E stain, original magnification 400x.

Sixty days after parturition, on June 9, 2017, the cow was artificially inseminated, and pregnancy was confirmed by ultrasonography on July 11, 2017. However, she aborted spontaneously on December 3, 2017, at 177 days (~6 months) of gestation. The aborted female fetus and a sample of placenta collected at the time of abortion, were subjected to gross pathologic examination. Samples of fetal brain, heart, kidney, and placenta were collected and preserved in a freezer at −20°C until processed by PCR for *N. caninum* DNA detection (see below). Additionally, samples of fetal tissues and placenta were immersion-fixed in 10% neutral buffered formalin for 48–72 h, routinely processed, embedded in paraffin and microtome-sectioned to produce 4- to 5-μm-thick sections that were stained with hematoxylin and eosin (H&E) for histopathology. Microscopic examination revealed typical lesions of *N. caninum* abortion (10, 11), including multifocal moderate non-suppurative necrotizing encephalitis with gliosis, multifocally extensive lymphocytic and histiocytic myocarditis, skeletal and glossal myositis (Figure 1), multifocal lymphocytic and histiocytic portal hepatitis, and severe extensive necrotizing lymphocytic and histiocytic placentitis (Figure 2) with trophoblastic necrosis, chorionic edema, and multifocal mineralization. The results of the histopathologic examination of the aborted fetus and placenta indicated that the infecting *N. caninum* (see below) had pathogenic and abortigenic potential under natural conditions.

Frozen fetal brain, heart, kidney, and placenta were processed for DNA extraction and *N. caninum* detection by PCR amplification as previously described (9), with positive results in all samples. Microsatellite typing performed as previously described (9, 12) allowed to amplify eight of nine targeted microsatellites of the *N. caninum* detected in the placenta and/or tissues of the aborted fetus (Table 1). Of the nine targeted microsatellites (MS4, MS5, MS6A, MS6B, MS7, MS8, MS10, MS12, and MS21), only 1 (MS7) could not be amplified from any of the fetal tissues or placenta. The microsatellite typing indicates that seven of these genetic markers are shared with isolate NcUru3 (Table 1), 6 (MS5, MS6B, MS8, MS10, MS12 and MS21) are shared with isolate NcUru4, 5 (MS5, MS8, MS10, MS12 and MS21) are shared with isolate NcUru1, and 2 (MS6A and MS8) are shared with isolate NcUru2 (9). Marker MS6A is only shared with *N. caninum* NcUru2, but not with NcUru1, NcUru3, nor NcUru4. Altogether these results indicate that the genotype infecting the aborted fetus is distinct from NcUru3, isolated from the newborn calf delivered by the same dam after a successful completion of her first gestation, ~8 months before the abortion, at least at the MS6A level. The abortion-causing genotype is also distinct from NcUru1, NcUru2, and NcUru4, isolated from congenitally infected dairy calves in Uruguay, as well as from other strains characterized from aborted dairy fetuses in this country (9). While the genetic difference detected only at the level of MS6A could be regarded as a minor difference between strains, the results confirm that they are not genetically identical, and they should be considered de facto different genotypes even if they have a common ancestor or one evolved from the other. Whether these strains have additional differences in other genomic regions was not explored, as sequencing of DNA extracted from spontaneously aborted fetuses is challenging mostly due to DNA preservation/quality issues.

**Table 1:** Pregnancy outcome, dates and results of microsatellite typing in two strains/genotypes of *N. caninum* infecting the offspring of the Holstein dam.

**Pregnancy outcome (gestation)**	**Date**	**Sample**	**Microsatellite markers**								
			**MS4**	**MS5**	**MS6A**	**MS6B**	**MS7**	**MS8**	**MS10**	**MS12**	**MS21**
Delivery of a viable healthy calf (1st gestation)	Apr. 10, 2017	DNA extracted from *N. caninum* isolate NcUru3	13	14	15	12	9.1	13	6.15.9	16	6
Abortion at 177 days of gestation (2nd gestation)	Dec. 3, 2017	DNA extracted from fetal brain (sample 1)	13	14	12	12	NI	NI	NI	NI	NI
		DNA extracted from fetal brain (sample 2)	13	NI	12	12	NI	13	NI	16	6
		DNA extracted from fetal heart	13	NI	NI	12	NI	13	NI	NI	6
		DNA extracted from fetal kidney	NI	NI	NI	NI	NI	13	NI	NI	NI
		DNA extracted from placenta	NI	NI	12	NI	NI	NI	6.15.9	16	6

*NI, not identified*.

To our knowledge, this is the first description of one same dam transmitting two different genotypes/strains of *N. caninum* to her offspring in subsequent gestations, which represents a relevant contribution to the current knowledge of the molecular epidemiology of neosporosis under non-experimental conditions. Interestingly, natural infection with *N. caninum* strain NcUru3, which resulted in congenital transmission in the first successful gestation, did not confer protection against spontaneous abortion caused by the different *N. caninum* genotype in the subsequent gestation. To assess whether the dam had IgG antibodies recognizing NcUru3 at the time of abortion, a serum sample was processed by immunoblotting (13) against a lysate of NcUru3 tachyzoites. A serum sample obtained of the calf from which this isolate originated collected before ingestion of colostrum was processed in parallel. Both animals had serum IgG recognizing NcUru3, although this humoral immune response in the dam did not prevent the other genotype from infecting its fetus and inducing fetoplacental lesions and abortion. Noteworthy, when inoculated in mice, *N. caninum* isolate NcUru3 was less virulent than *N. caninum* reference strain Liverpool (14). Although many factors may have influenced the clinical outcome in the dam studied in this work, this lower relative virulence of NcUru3 would be consistent with the outcome of her first gestation which resulted in the delivery of a viable calf.

Little information is available on clinical outcomes in pregnant cattle infected with different *N. caninum* strains over time, and while the results of our report do not allow to draw major conclusions in this regard, the available information will be discussed here. In a study conducted in Argentina, four seropositive cows naturally exposed to non-characterized strain(s) of *N. caninum* were later experimentally inoculated intravenously with tachyzoites of a presumably different strain (NC-6 Argentina) at 65 days of gestation (15). One of the cows aborted but the fetus was not available for examination, thus the cause of the abortion and the infection status of the fetus were not further investigated. The remaining three non-aborted cows were sent to slaughter and their fetuses were retrieved at 108 ± 2 days of gestation. All three fetuses had histologic lesions compatible with *N. caninum* infection, and multilocus microsatellite analysis revealed that NC-6 Argentina was the infecting strain (15). The study demonstrated the ability of intravenously inoculated tachyzoites of strain NC-6 Argentina to cross the placenta, infect the fetus, and induce fetal pathology in seropositive cattle presumably exposed to a different strain. Our results further support the idea that under natural circumstances different *N. caninum* genotypes can cross the placenta and infect the offspring in a single dam over time and in subsequent gestations. However, whether the clinical outcome varies with the infecting genotype should be further explored, ideally in controlled experimental conditions. Our results also suggest that natural exposure to one strain does not necessarily protect against abortion by other genotypes in subsequent gestations, even in the face of maternal IgG specifically recognizing the previous infecting strain at the time of abortion. After aborting in her second gestation, the cow in our study was sent to the slaughterhouse so no additional follow-up was conducted.

How the cow included in this study acquired the infection with either genotype is unknown. She may have been born infected with NcUru3 or acquired the infection at any time point in her postnatal life before delivering her first normal calf. Similarly, whether she was already infected with the other genotype at the time of her first calving, or whether she acquired this infection in the period elapsed between the first normal calving and the abortion in the second gestation is unknown. Moreover, whether the aborted fetus acquired the transplacental infection after an episode of endogenous reactivation or an event of exogenous transmission is also unknown. Different scenarios such as the occurrence of a coinfection (concurrent infection with both genotypes), sequential infection with different genotypes across time, or even a mutation taking place in NcUru3 at least at the level of MS6A and giving rise to a different genotype between the first calving and abortion seem all plausible. It is worth mentioning that various *N. caninum* isolates (notably NcUru 1, 2 and 3) are known to circulate in the same farm where this dam delivered the viable calf after completion of her first pregnancy and aborted in the following pregnancy [(9), and authors personal observation]. In this context, exposure to multiple strains is likely.

Although factors that may determine clinical outcomes in natural cases of neosporosis in cattle are numerous, including the gestational age at the time of fetal infection (i.e., the infection and clinical outcome can be fatal in fetuses infected in the second trimester of gestation while subclinical in fetuses infected in the third trimester), transmission pathways to the fetus (exogenous vs. endogenous transplacental transmission), previous exposure, immunity, the number of pregnancies (nulliparous vs. multiparous dams) and other factors of the dam, herd, and environment (11, 16), it is believed that many factors remain unknown (11). In this context, the eventual role of genetic determinants of the infecting *N. caninum* strain in clinical outcome should not be neglected and deserves further research. The results of the present work could be useful for researchers working in the development of *N. caninum* vaccines, particularly those based on a single strain/genotype.

## Data availability statement

The original contributions presented in the study are included in the article/supplementary material, further inquiries can be directed to the corresponding author/s.

## Ethics statement

The animal study was reviewed and approved by an Ethics Committee for the Use of Animals in experimentation (CEUA) at INIA (protocol # 2017.1).

## Author contributions

FG and VA conceptualized the study and performed the autopsy and postmortem sampling of the newborn calf. FG wrote the first draft of the manuscript, performed the histopathologic examination of the fetus and placenta, and obtained microphotographs. VA, CS, and FG performed serum sampling. CS performed ELISA testing. MF, CR, and AC conducted molecular testing and analyses and immunoblotting. All authors contributed to manuscript revision, read, and approved the submitted version of the manuscript.

## Funding

This work was funded by grants FSSA_X_2014_1_105696 and FSSA_X_2014_1_106026 of the Uruguayan Agencia Nacional de Investigación e Innovación (ANII), and grant PL_27 N-23398 of the Instituto Nacional de Investigación Agropecuaria (INIA). FG, CS, MF, CR, and AC are members of the Sistema Nacional de Investigadores (SNI) of ANII.

## Conflict of interest

The authors declare that the research was conducted in the absence of any commercial or financial relationships that could be construed as a potential conflict of interest.

## Publisher's note

All claims expressed in this article are solely those of the authors and do not necessarily represent those of their affiliated organizations, or those of the publisher, the editors and the reviewers. Any product that may be evaluated in this article, or claim that may be made by its manufacturer, is not guaranteed or endorsed by the publisher.
